# Rainfall driven and wild-bird mediated avian influenza virus outbreaks in Australian poultry

**DOI:** 10.1186/s12917-021-03010-9

**Published:** 2021-09-14

**Authors:** Marta Ferenczi, Christa Beckmann, Marcel Klaassen

**Affiliations:** 1 Centre for Integrative Ecology, School of Life & Environmental Sciences, Deakin University, 75 Pigdons Road, 3216 Geelong, VIC Australia; 2 School of Science, Western Sydney University, Locked Bag 1797, 2751 Penrith, NSW Australia; 3 Hawkesbury Institute for the Environment, Western Sydney University, Locked Bag 1797, 2751 Penrith, NSW Australia

**Keywords:** waterfowl, infection dynamics, climatic forcing, domestic chicken, domestic duck, *Gallus gallus domesticus*, *Anas platyrhynchos domesticus*, human-wildlife conflict

## Abstract

Globally, outbreaks of Avian Influenza Virus (AIV) in poultry continue to burden economies and endanger human, livestock and wildlife health. Wild waterbirds are often identified as possible sources for poultry infection. Therefore, it is important to understand the ecological and environmental factors that directly influence infection dynamics in wild birds, as these factors may thereby indirectly affect outbreaks in poultry. In Australia, where large parts of the country experience erratic rainfall patterns, intense rainfalls lead to wild waterfowl breeding events at temporary wetlands and increased proportions of immunologically naïve juvenile birds. It is hypothesized that after breeding, when the temporary wetlands dry, increasing densities of immunologically naïve waterbirds returning to permanent water bodies might strongly contribute to AIV prevalence in wild waterfowl in Australia. Since rainfall has been implicated as an important environmental driver in AIV dynamics in wild waterbirds in southeast Australia and wild waterbirds are identified globally to have a role in virus spillover into poultry, we hypothesise that rainfall events have an indirect effect on AIV outbreaks in poultry in southeast Australia. In this study we investigated this hypothesis by examining the correlation between the timing of AIV outbreaks in poultry in and near the Murray-Darling basin in relation to temporal patterns in regional rainfall since 1970. Our findings support our hypothesis and suggest that the risk of AIV outbreaks in poultry increases after a period of high rainfall, with peak AIV risk two years after the onset of the high-rainfall period. This is presumably triggered by increased rates of waterbird breeding and consequent higher proportions of immunologically naïve juvenile waterbirds entering the population directly after major rainfall events, which subsequently aggregate near permanent water bodies when the landscape dries out.

**Supplementary Information:**

The online version contains supplementary material available at 10.1186/s12917-021-03010-9.

## Introduction

High pathogenicity outbreaks of Avian Influenza Virus (AIV) in domestic poultry and the possibility of transmission of AIV to humans can result in extensive socio-economic costs [1–3]. AIV in its low pathogenicity form (typically causing only mild or non-detectable clinical signs in poultry; termed LPAI) occurs naturally in wild bird populations [4]. In recent years, research has focused on the wild-bird assisted dispersal of high pathogenicity forms (typically causing severe clinical signs and rapid death in gallinaceous poultry; termed HPAI) and notably AIV-H5-Clade-2.3.4.4, of which wave upon wave currently causes havoc in poultry industries across the globe e.g. [5, 6]. Another role for wild birds in the infection of poultry results from the occasional evolution of a HPAI in poultry after alleged exposure to LPAI from wild birds [7–9]. Virus spillover onto poultry farms could occur when infected wild birds enter or come close to poultry barns. These wild birds could either directly infect the chickens, ducks and other poultry species, or indirectly contaminate water and surfaces from where the virus is transmitted to the poultry, potentially assisted by farm workers, pets or transport of equipment [10, 11]. Therefore, we can assume that the same ecological and environmental factors that foster epizootics of LPAI in wild birds would indirectly result in increased incidence of LPAI (and subsequently evolved HPAI) outbreaks in poultry.

Due to HPAI outbreaks in poultry there is great interest in the ecological and environmental factors that influence infection dynamics in wild birds and the possible virus transmissions between wild birds and poultry e.g. [12]. To date, the majority of research on AIV dynamics in wild birds has been conducted in ducks in the genus *Anas*, being the prime wild bird reservoir of AIV [13, 14], and in the northern hemisphere. The AIV pattern observed there is strongly seasonal, with a yearly peak in late summer/early autumn, followed by low prevalence in winter [15, 16]. Across North America the intensity of these infection dynamics varies geographically in relation to the strength of the seasonal patterns which is thought to drive recruitment patterns [17]. Studies that included birds from the southern hemisphere showed that peaks of AIV prevalence in waterfowl communities are lower there than in the northern hemisphere [18]. Nonetheless, a shallow seasonal peak was suggested in southern hemisphere birds [19]. Furthermore, recent studies in temperate southeast Australia [18], where dabbling ducks are also identified as the primary AIV reservoir [20], showed that AIV prevalence was related to irregular, non-seasonal rainfall patterns.

Several ecological mechanisms have been studied as potential drivers of AIV dynamics in wild birds [16, 17, 19, 21]. Among wild waterbird communities, three ecological mechanisms have been suggested as the primary drivers of the seasonal AIV dynamics in the northern hemisphere: (i) the annual congregation of migratory birds at staging and wintering sites increases contact rates between individuals, and thereby infection rates [19], (ii) an increase in the abundance of immunologically naïve young birds results in a higher number of individuals susceptible to infection in the waterbird community [16, 22] and (iii) increases in energy-demanding activities, notably in relation to migration, potentially impairing immunocompetence [21, 23]. In general, the ecological drivers for disease dynamics are importantly linked to seasonal variation in resources in the northern hemisphere [21, 24, 25]. Large parts of the globe, however, are far less seasonal [26].

In Australia, for instance, water availability is highly variable and an important factor in the ecology and stark variations in numbers of waterfowl [27–29]. Across much of the Australian continent, climatic conditions are extreme and non-seasonal [30]. Although regular rainfall occurs seasonally in the Australian tropics (summer) and the temperate southeast and southwest regions (winter-spring), water availability is largely non-seasonal across the rest of the continent [30, 31]. In southeastern Australia, inter-annual variation in rainfall is very high, with higher rainfall being positively related to waterfowl breeding [31]. Wet and dry periods can each persist for several years [31], occasionally creating extreme climate events, such as the ‘Big Dry’ phenomenon in southeastern Australia between 1997 and 2009 [32].

Globally, waterfowl numbers have been found to be tightly linked to water availability in the landscape e.g. [33] and it is thus unsurprising that these irregular rainfall patterns in Australia strongly influence the movement and breeding biology of many Australian waterfowl species. During wet periods, bird numbers increase at flooded areas where food sources become available, creating appropriate conditions for breeding [34, 35]. Afterwards, when flooded areas start to dry and reduce in size, waterbirds congregate on the remaining wetlands [36–38]. Klaassen et al. [39] suggested that the non-seasonal and often multi-year alternations of wet and dry periods that influence the breeding ecology of waterfowl might, in turn, affect the temporal pattern of AIV prevalence on the Australian continent. Applying the previously mentioned ecological drivers (i.e. i, ii, iii) to the climatic conditions in the southern hemisphere, Klaassen et al. [39] hypothesized that intense rainfall leads to breeding events and increased numbers of immunologically naïve juvenile birds. After breeding, when the temporary wetlands dry, increasing densities of immunologically naïve waterbirds returning to permanent water bodies might importantly influence AIV prevalence in wild waterfowl in Australia. In addition, the reduced food availability that accompanies the drying ephemeral wetlands can lead to reduction in birds’ immunocompetence [40] and therefore further increase AIV infection risk. Ferenczi et al.’s [18] findings from temperate southeast Australia also support Klaassen et al.’s [39] hypothesis that irregular rainfall influences population dynamics and age structure within the duck community, which may subsequently affect AIV dynamics.

As (1) rainfall is an important environmental driver in AIV dynamics in wild Australian waterbirds [18] and (2) wild waterbirds, especially ducks, are identified globally to have a role in virus spillover into poultry [41–43] and (3) all HPAI poultry outbreaks in Australia can be traced back to an endemic Australian H7 lineage most likely spilled over from wild birds [44], we suggest that rainfall events have an indirect effect on AIV outbreaks in Australian poultry. We investigated this hypothesis by examining the correlation between the timing of AIV outbreaks in poultry in a region that contains most of Australia’s poultry-dense areas and accounts for most of Australia’s poultry production, the Murray-Darling basin and nearby locations, in relation to temporal patterns in regional rainfall.

## Materials and methods

### Avian influenza virus outbreak data

The Australian poultry industry is dominated by the production of chicken eggs and meat, 60 % of which is produced within the Murray-Darling Basin. We tabulated the LPAI and HPAI outbreaks on poultry farms in Australia from the National Avian Influenza Surveillance Dossier [45], NSW Animal Health Surveillance Newsletters [46], Animal Health in Australia Annual Reports [47], World Animal Health Information Database (WAHID) Interface [48] and reports in the primary literature [49–51]. There is no systematic surveillance for AIV within the Australian poultry industry and the majority, if not all, LPAI and HPAI detections were made upon investigating clinical signs, which, albeit mild, often also arise upon infection with LPAI. However, detections of HPAI do trigger increased vigilance and the two novel infections of LPAI within Victoria, one month after a HPAI outbreak in this state, might have gone undetected if that HPAI outbreak had not occurred. We found that, with one exception in Tasmania and one in Western Australia [52], all the AIV outbreaks in poultry in Australia occurred in or in close proximity to the Murray-Darling basin (i.e. within 100 km of the basin’s boundary). On a continental scale, the Murray Darling Basin is a highly significant waterbird area including as many as 18,500 interconnected wetlands of which 98 support more than 10,000 waterbirds each, notably ducks [53]. Also, from a poultry-production perspective the area is of continental significance, which is the prime rationale for this study to focus on this basin. In our analysis, we thus included all outbreaks in commercial poultry from the Murray-Darling basin and sites within 100 km of its boundary. Outbreaks and their timing of first occurrence (i.e. month and year) were defined as events with occurrence of severe (in case of HPAI) or mild (in case of LPAI) clinical signs. In cases where multiple farms were infected with the same strain only the initial outbreak was included to capture spillover from wild birds and not farm-to-farm transmission.

### Weather data and statistical analysis

In order to investigate AIV outbreak events in relation to rainfall, we obtained monthly total rainfall data (mm) averaged across the entire Murray-Darling basin from the Australian Bureau of Meteorology [54] between January 1970 until October 2020. The effects of weather are not always immediately expressed in ecological processes [28, 55], thus there may be a cumulative effect and/or a time lag between rainfall, waterfowl breeding events and associated changes in the epidemiology of AIV within wild bird populations and AIV outbreaks in poultry. Ecological meaningful rainfall variables that can potentially predict AIV outbreaks in poultry may thus vary in (1) the period over which rainfall is integrated (termed “rainfall period” from here on) and (2) the time lag between this rainfall period and the increased AIV prevalence after breeding that ultimately leads to an increased risk of AIV outbreaks in poultry (termed “time lag period” from here on). We tested 600 models using logistic regression in R [56], where for each month from January 1970 to October 2020 we used presence/absence of an outbreak as the response variable and total monthly rainfall as the explanatory variable. Each of the 600 models varied in how rainfall was calculated, with the rainfall period varying one to 24 months and the time lag period varying from zero to 24 months. For example, the rainfall category of two months “rainfall period” with zero “time lag period” means that total monthly rainfall was averaged over two months preceding a focal month. As another example, for a rainfall category of three months “rainfall period” with one month “time lag period” means that total monthly rainfall was averaged over the second, third and fourth month (i.e. skipping the first month) prior to the focal month. To appropriately weight incidentally co-occurring outbreaks (i.e. outbreaks happening within the same month of the same year; 3 occurrences), one of the outbreaks was moved to the following month. To select the best model(s) among the 600 tested, we used Akaike’s Information Criterion (AIC) considering the top model to be the model with the lowest AIC but models within 2 AIC units also to have substantial support.

## Results

**Fig. 1:**
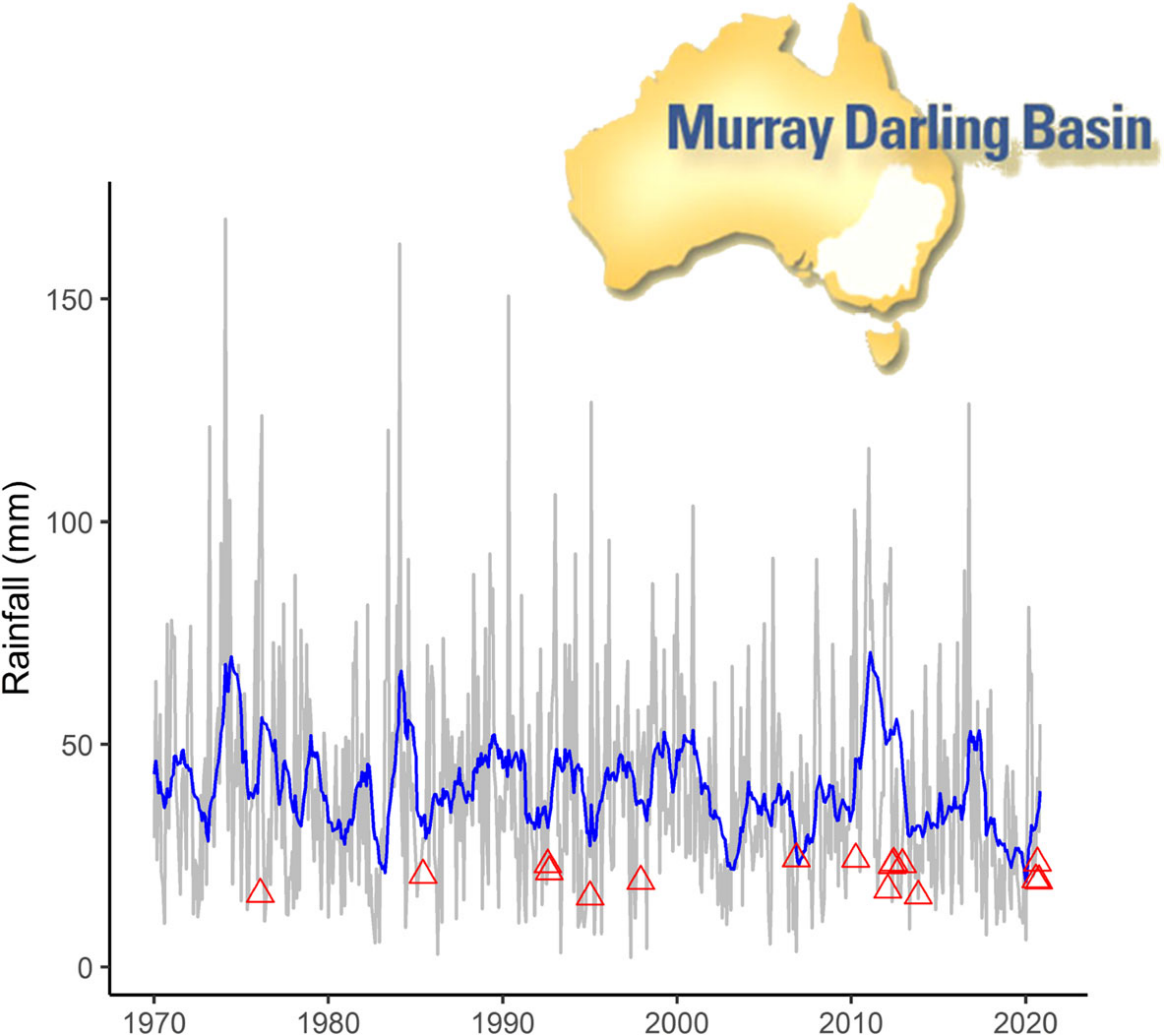
Timeline of avian influenza outbreaks (red triangles) in commercial poultry in the Murray-Darling basin and close vicinity against mean monthly (grey line) and right-aligned rolling mean rainfall across the basin (blue line; i.e. indicating the mean rainfall in the preceding twelve-months)

**Fig. 2:**
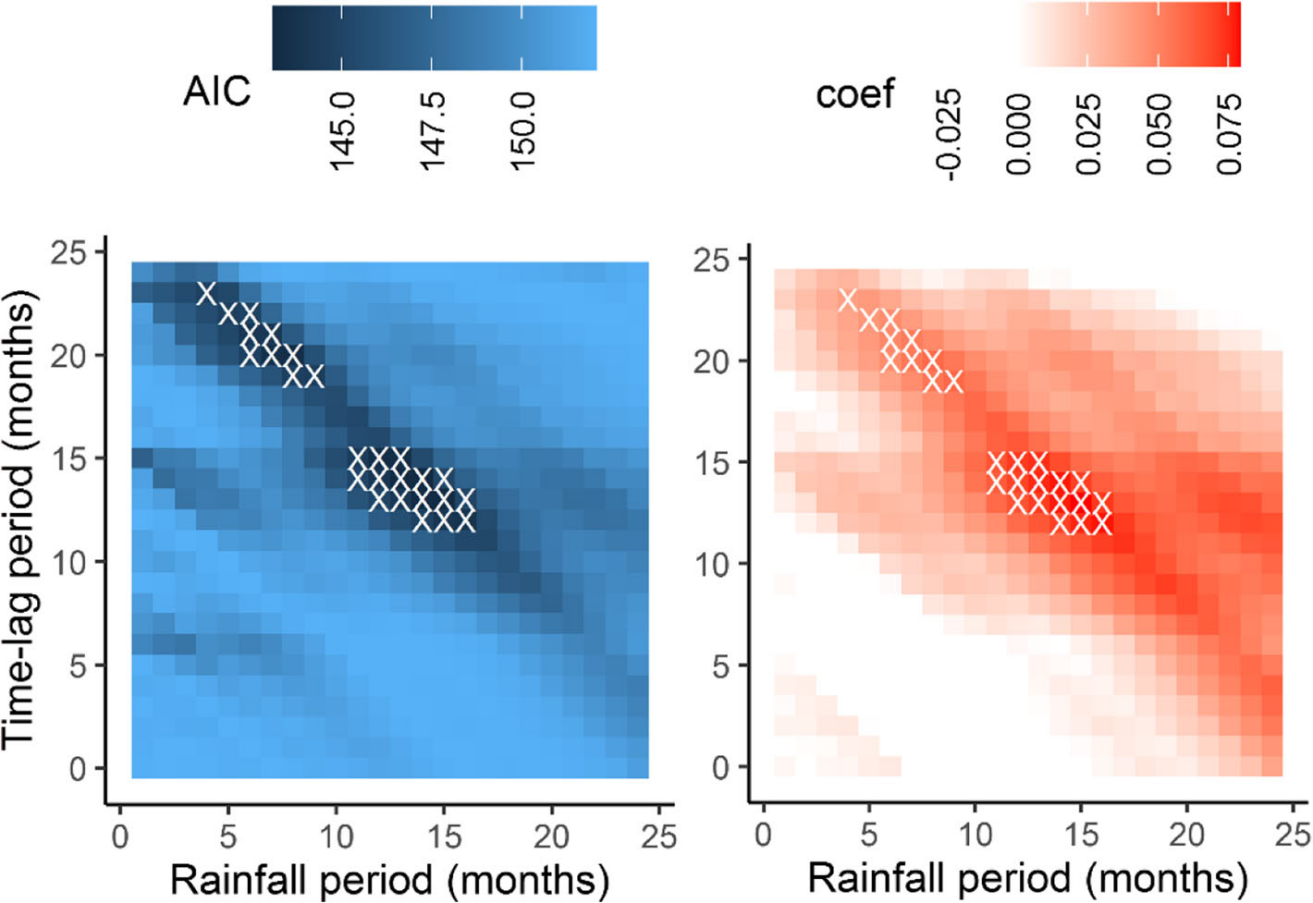
Heat maps of AIC index (left panel) and log odds slopes (right panel) as a function of rainfall period (x-axis) and time-lag period (y-axis) with the 26 combinations resulting in model outcomes with the lowest AIC values (i.e. within 2 AIC units) marked with a white ×. Lower AIC values (i.e. darker blue shading) identify models that better predict AIV outbreaks in poultry from rainfall data. Higher log odds slope coefficients (i.e. darker red shading) indicate a stronger positive relationship between rainfall and AIV outbreak risk

We found eight HPAI and eight LPAI outbreaks linked to unique strains in commercial poultry across the Murray-Darling basin and close vicinity between 1976 and 2020 to analyse AIV outbreak events in relation to rainfall (Fig. 1; Table 1). For the 610 months over the period January 1970 to October 2020, 600 different rainfall indices were calculated varying in rainfall and time-lag period. The best model with the lowest AIC (143.11) predicted that the likelihood of an outbreak would increase with increased rainfall (slope of the log odds 0.08, P < 0.002). The rainfall index for this top model was calculated over a period of 14 months and had a subsequent time-lag period of also 14 months. Thus, according to this model, outbreaks tended to occur 28 months after the onset of a 14-month rainfall period. An overview of the estimated AICs and log odds slopes across all 600 tested models are depicted in Fig. 2. Another 25 models where within 2 AIC units of the top model, all with a positive slope of the log odds between 0.04 and 0,08 (in all cases P < 0.007) and should thus also be considered as good candidate models (Fig. 2). Fourteen of those had similar rainfall (11–16 months) and time-lag (12–15 months) periods to the best model. Ten had considerably shorter rainfall periods (5–9 months) but longer time-lag periods (19–23 months). Remarkably though, all 26 models tended to predict a higher chance of AIV outbreaks in poultry around 27 months after the onset of a particularly high rainfall period (i.e. the sum of rainfall and time-lag period has a median of 27 months and a range of 25–29 months and the top models thus all fall along a diagonal in both panels of Fig. 2).

**Table 1:** High pathogenicity (HPAI) and low pathogenicity (LPAI) avian influenza virus outbreaks in commercial poultry across the Murray-Darling basin and close vicinity between 1976 and 2020

Year	Month	State	Location	Affected stock	HPAI / LPAI subtype	Reference
1976	January	Victoria	Keysborough (outer suburbs of Melbourne)	chicken, duck	HPAI, H7N7	[45]
1985	May	Victoria	Bendigo	chicken	HPAI, H7N7	[45]
1992	July	Victoria	West Victoria	duck	LPAI, H3N8	[51]
1992	July	Victoria	Bendigo	chicken, duck	HPAI, H7N3	[45, 49]
1994	December	Queensland	Lowood	chicken	HPAI, H7N3	[45]
1997	November	New South Wales	Tamworth	chicken, emu	HPAI, H7N4	[45, 50]
2006	October	New South Wales	Sydney Basin	chicken, duck	LPAI, H6N4	[45]
2010	March	New South Wales	Sydney basin	chicken	LPAI, H10N7	[46]
2012	January	Victoria	Melbourne	duck	LPAI, H5N3	[47, 48]
2012	April	New South Wales	Hunter Valley	turkey	LPAI, H9N2	[46]
2012	April	New South Wales	North Coast	duck	LPAI, H4N6	[46]
2012	November	New South Wales	Hunter Valley	chicken	HPAI, H7N7	[46]
2013	October	New South Wales	Young	chicken	HPAI, H7N2	[48]
2020	July	Victoria	Lethbridge	chicken	HPAI, H7N7	[48]
2020	August	Victoria	Lethbridge	turkey	LPAI, H5N2	[48]
2020	August	Victoria	Kerang	emu	LPAI, H7N6	[48]

## Discussion

The top models in our analysis indicated that the average monthly rainfall was significantly higher prior to outbreaks when allowing for time lags of 12 to 23 months (14 months in the best model). These findings support our initial hypothesis and suggest that an increased risk of AIV outbreaks in poultry exists after (1) a period of intense rainfall over a period of four to 16 months (14 months in best model), presumably triggering increased waterbird breeding and increased numbers of immunologically naïve juvenile waterbirds, which (2) enter the population at gradually increasing densities over a time lag between 12 and 23 months (14 months in best model). The time lag between intense rainfalls and outbreaks in poultry are in agreement with Ferenczi et al.’s [18] findings from southeast Australia, where higher rainfall three to seven months before higher AIV prevalence in wild waterbird populations was observed. That the time lag is substantially longer in the present study than in Ferenczi et al. [18] probably relates primarily to the extra time needed for AIV to spillover from wild birds into poultry, which may importantly be facilitated by increased densities of ducks following a dry period (see below) and possibly, at least in half of the cases, the time needed for the LPAI to evolve in a HPAI.

A dominant feature of Australian climate is the ENSO-linked irregularity in both timing and location of wet and dry periods [37, 57, 58]. These erratic climate patterns may relax seasonality in waterfowl breeding, where reproduction occurs after periods of higher rainfall and associated increases in food availability [28, 29, 34]. Ferenczi et al. [18] indicated that after rainfall-triggered breeding events, the influx of juveniles that arrive from inland areas that mix with locally hatched juveniles, were likely drivers of AIV prevalence dynamics in two wild duck species on a coastal permanent wetland. Thus the time lag that is observed between breeding and the increased AIV prevalence in waterfowl populations after breeding [18] is likely reflected in AIV outbreaks in poultry.

The delay between a high rainfall period and the occurrence of AIV outbreaks in poultry is consistent with the earlier hypothesised idea that the likelihood of AIV outbreaks in poultry increases as temporary wetlands dry up [39]. As inland wetland systems contract with the onset of dry periods, associated wild waterbirds from these regions [34, 35] may concentrate on a few remaining waterbodies, such as farm dams. In these situations, they may be in direct or indirect contact with poultry, which increases the likelihood of virus transmission between wild and domestic birds.

Recognizing the role of rainfall as a major driver of waterbird population dynamics, Vijaykrishna et al. [59] showed a decrease in AIV diversity during years when the rainfall across Australia was below average. To our knowledge, Vijaykrishna et al.’s [59] rainfall driven evolutionary dynamics of AIV and Ferenczi et al.’s [18] rainfall driven viral prevalence in waterfowl are the only studies that address the idea that non-seasonal rainfall patterns are a major driver of AIV dynamics in this part of the world. Although rainfall is considered to be of less importance in AIV dynamics in the northern hemisphere, a few studies have found it to influence AIV prevalence in wild and domestic birds [60, 61]. East et al.’s [62] study of H5N1 HPAI infection risk analysis in Australia suggested that the areas of highest risk for introduction of AIV from wild birds into poultry were in eastern Australia where there are (1) higher densities of poultry farms; (2) more wetland habitats for waterbirds and (3) the climate is wetter [62].

Our study, albeit drawing conclusions based on correlations exclusively, highlights the importance of investigating AIV dynamics in both wild and domestic birds in relation to different environmental and ecological factors, allowing for a better understanding of AIV transmission risks between them. Additionally, and notably for systems regularly experiencing extreme weather events, such studies may allow for an improved understanding of the climatic drivers of disease dynamics. Climatic forcing of disease dynamics is commonly assumed for both humans and wildlife [17, 24, 63]. However, the evaluation of causality between weather conditions and disease patterns is often hampered by high levels of seasonality, which notably prevail in the northern hemisphere. Disease-dynamics studies in regions of the world with less predictable climatic conditions, such as in large parts of Australia and many other areas of the southern hemisphere, may thus provide important insights in the true drivers of disease dynamics and the consequences of climate change on disease dynamics [47, 64].

## Supplementary information

**Additional file 1**

## Data Availability

The data that support the findings of this study are available from the Australian Bureau of Meteorology (http://www.bom.gov.au/climate/change/index.shtml#tabs=Tracker&tracker=timeseries); NSW Animal Health Surveillance Newsletters (https://www.dpi.nsw.gov.au/about-us/publications/animal-health-surveillance); Animal Health in Australia Annual Reports (www.animalhealthaustralia.com.au); and World Animal Health Information Database (WAHID) Interface (www.oie.int/wahis_2/public/wahid.php/Wahidhome/Home). Additional file 1 is provided as an Rmarkdown html file, containing the R script and detailed output of the calculations.
