# Supplementary material for: Rainfall driven and wild-bird mediated avian influenza virus outbreaks in Australian poultry
Source: BMC Vet Res. 2021 Sep 14;17:306. doi: 10.1186/s12917-021-03010-9 (PMC8439068; doi:10.1186/s12917-021-03010-9)
Supplement: Supplementary file 1 — Additional file 1 [file 12917_2021_3010_MOESM1_ESM.html]

Rainfall as driver of Australian AIV outbreaks


Code 

- Show All Code
- Hide All Code

# Rainfall as driver of Australian AIV outbreaks

#### Marcel Klaassen

#### 30 July 2021

```
knitr::opts_chunk$set(echo = TRUE)

library(jtools)
library (performance) #for pseudo R2
library(ggplot2)
library(tidyr) #for long format
library(zoo) #for running mean

try(setwd("E:/OneDrive - Deakin University/Active projects/Marti"))

#read MDB rainfall data downloaded from BOM
R <- read.csv("latest.22233.csv")
#select all years past 1965
R <- R[which(R$Year>1965),]
#make long format
S <- gather(R,key="Month",value="rainfall",-Year)   #uses tidyr
S$month <- match(S$Month,month.abb)                 #recode month to numbers
S <- S[order(c(S$Year,S$month)),]
S <- S[which(!is.na(S$rainfall)),]                  #remove missing cases

#The outbreak dataframe
O <- data.frame(Year =c(1976, 1985, 1992, 1992, 1994, 1997, 2006, 2010, 2012, 2012, 2012, 2012, 2013, 2020, 2020, 2020),
                month=c(   1,    5,    7,    8,   12,   11,   10,    3,    1,    4,    5,   11,   10,    7,    8,    9),
                   HL=c(  "H",  "H",  "L",  "H",  "H",  "H",  "L",  "L",  "L",  "L",  "L",  "H",  "H",  "H",  "L",  "L"))
O$outbreak <- 1

I <- merge(S,O, by=c("Year","month"), all.x=TRUE)
I$outbreak[which(is.na(I$outbreak))] <- 0
I <- I[!duplicated(I),]   #remove months where there are multiple outbreaks in 1 month
```

## Outbreaks in poultry in relation to rainfall

The underlying rainfall data are the monthly rainfall data across the entire Murray Darling Basin up till and including October 2020, which were downloaded from http://www.bom.gov.au/climate/change/index.shtml#tabs=Tracker&tracker=timeseries&tQ=graph%3Drain%26area%3Dmdb%26season%3Dallmonths%26ave\_yr%3D0

### Rainfall period

The number of months across which rainfall is being averaged

### Time-lag period

The number of months following the **rainfall period** until the outbreak. It is assumed that after a major rainfall period

- it takes a while for naive ducks to enter the population,
- for the environment to dry out
- for birds to start aggregating
- for an epizootic to evolve
- once spill over of LPAI from wild birds into poultry has taken place for a HPAI to evolve

## Statistics

We used generalised linear modelling to conduct a logistic regression across all months from January 1970 until October 2020, where we scored an outbreak month as 1 and a non-outbreak month as 0 as dependent variable.

To appropriately weight co-occurring outbreaks (i.e. outbreaks happening within the same months of the same year), one of the outbreaks was moved to the following month.

We ran 600 models where in each model we used rainfall as the explanatory variable calculated for a different combination of **rainfall period** and **time-lag period**.

Below the output of the best model is printed

```
#define the output df
M <- data.frame(AIC = numeric(700))
M$r2 <- NA
M$coef <- NA
M$RainfallPeriod <- NA
M$TimeLagPeriod <- NA
M$N <- NA
i <- 0

for (r in 1:24) {      #number of rainfall months
  for (t in 0:(24)) {    #time-lag months
  i <- i+1
  I$rainmean <- NA
  for (j in (r+t+1):nrow(I)) {
    I$rainmean[j] <- mean(c(I$rainfall[(j-t-r):(j-t-1)]))
   }
  model <- glm(outbreak ~ rainmean, data=I[which(I$Year %in% c(1970:2020)),],family=binomial)
  #summ(model)
  M$AIC[i] <- model$aic
  M$r2[i] <- r2(model)$R2_Tjur
  M$coef[i] <- model$coefficients[2]
  M$N[i] <- length(model$residuals)
  
  #get the rainfall and time-lag periods
  M$RainfallPeriod[i] <- r
  M$TimeLagPeriod[i] <- t
  }
}

#arrange df starting with lowest AIC
M <- M[order(M$AIC),]
M <- M[which(!is.na(M$r2)),]
#make a marker for top models
M$top <- ifelse(M$AIC <= min(M$AIC)+2,"X","")

#how many top models do we have?
TOP <- length(M$top[which(M$top=="X")])

#make variable to store TOP model predictions
#add fitted values from best model as Expected Probabilities
I$fit <- NA
I$fit[48:658] <- 0


#run best models
for (i in 1:TOP) {
 I$rainmean <- NA
 r <- M$RainfallPeriod[i]
 t <- M$TimeLagPeriod[i]

 for (j in (r+t+1):nrow(I)) {
    I$rainmean[j] <- mean(c(I$rainfall[(j-t-r):(j-t-1)]))
   }
 model <- glm(outbreak ~ rainmean, data=I[which(I$Year %in% c(1970:2020)),],family=binomial)
 print(summary(model))
 I$fit[48:658] <- model$fitted.values + I$fit[48:658]
 }
```

```
## 
## Call:
## glm(formula = outbreak ~ rainmean, family = binomial, data = I[which(I$Year %in% 
##     c(1970:2020)), ])
## 
## Deviance Residuals: 
##     Min       1Q   Median       3Q      Max  
## -0.6469  -0.2493  -0.1950  -0.1589   3.2142  
## 
## Coefficients:
##             Estimate Std. Error z value Pr(>|z|)    
## (Intercept)  -7.0098     1.2070  -5.807 6.34e-09 ***
## rainmean      0.0778     0.0251   3.100  0.00194 ** 
## ---
## Signif. codes:  0 '***' 0.001 '**' 0.01 '*' 0.05 '.' 0.1 ' ' 1
## 
## (Dispersion parameter for binomial family taken to be 1)
## 
##     Null deviance: 148.08  on 609  degrees of freedom
## Residual deviance: 139.11  on 608  degrees of freedom
## AIC: 143.11
## 
## Number of Fisher Scoring iterations: 7
## 
## 
## Call:
## glm(formula = outbreak ~ rainmean, family = binomial, data = I[which(I$Year %in% 
##     c(1970:2020)), ])
## 
## Deviance Residuals: 
##     Min       1Q   Median       3Q      Max  
## -0.5779  -0.2480  -0.1943  -0.1591   3.2014  
## 
## Coefficients:
##             Estimate Std. Error z value Pr(>|z|)    
## (Intercept) -6.92039    1.17870  -5.871 4.33e-09 ***
## rainmean     0.07563    0.02440   3.100  0.00194 ** 
## ---
## Signif. codes:  0 '***' 0.001 '**' 0.01 '*' 0.05 '.' 0.1 ' ' 1
## 
## (Dispersion parameter for binomial family taken to be 1)
## 
##     Null deviance: 148.08  on 609  degrees of freedom
## Residual deviance: 139.13  on 608  degrees of freedom
## AIC: 143.13
## 
## Number of Fisher Scoring iterations: 7
## 
## 
## Call:
## glm(formula = outbreak ~ rainmean, family = binomial, data = I[which(I$Year %in% 
##     c(1970:2020)), ])
## 
## Deviance Residuals: 
##     Min       1Q   Median       3Q      Max  
## -0.5921  -0.2495  -0.1967  -0.1607   3.2115  
## 
## Coefficients:
##             Estimate Std. Error z value Pr(>|z|)    
## (Intercept) -7.07452    1.23151  -5.745 9.22e-09 ***
## rainmean     0.07944    0.02573   3.088  0.00202 ** 
## ---
## Signif. codes:  0 '***' 0.001 '**' 0.01 '*' 0.05 '.' 0.1 ' ' 1
## 
## (Dispersion parameter for binomial family taken to be 1)
## 
##     Null deviance: 148.08  on 609  degrees of freedom
## Residual deviance: 139.16  on 608  degrees of freedom
## AIC: 143.16
## 
## Number of Fisher Scoring iterations: 7
## 
## 
## Call:
## glm(formula = outbreak ~ rainmean, family = binomial, data = I[which(I$Year %in% 
##     c(1970:2020)), ])
## 
## Deviance Residuals: 
##     Min       1Q   Median       3Q      Max  
## -0.5800  -0.2495  -0.1956  -0.1593   3.1926  
## 
## Coefficients:
##             Estimate Std. Error z value Pr(>|z|)    
## (Intercept) -6.98777    1.20566  -5.796  6.8e-09 ***
## rainmean     0.07734    0.02509   3.083  0.00205 ** 
## ---
## Signif. codes:  0 '***' 0.001 '**' 0.01 '*' 0.05 '.' 0.1 ' ' 1
## 
## (Dispersion parameter for binomial family taken to be 1)
## 
##     Null deviance: 148.08  on 609  degrees of freedom
## Residual deviance: 139.20  on 608  degrees of freedom
## AIC: 143.2
## 
## Number of Fisher Scoring iterations: 7
## 
## 
## Call:
## glm(formula = outbreak ~ rainmean, family = binomial, data = I[which(I$Year %in% 
##     c(1970:2020)), ])
## 
## Deviance Residuals: 
##     Min       1Q   Median       3Q      Max  
## -0.6247  -0.2481  -0.1947  -0.1603   3.1939  
## 
## Coefficients:
##             Estimate Std. Error z value Pr(>|z|)    
## (Intercept) -6.87061    1.17788  -5.833 5.44e-09 ***
## rainmean     0.07456    0.02443   3.051  0.00228 ** 
## ---
## Signif. codes:  0 '***' 0.001 '**' 0.01 '*' 0.05 '.' 0.1 ' ' 1
## 
## (Dispersion parameter for binomial family taken to be 1)
## 
##     Null deviance: 148.08  on 609  degrees of freedom
## Residual deviance: 139.42  on 608  degrees of freedom
## AIC: 143.42
## 
## Number of Fisher Scoring iterations: 7
## 
## 
## Call:
## glm(formula = outbreak ~ rainmean, family = binomial, data = I[which(I$Year %in% 
##     c(1970:2020)), ])
## 
## Deviance Residuals: 
##     Min       1Q   Median       3Q      Max  
## -0.5687  -0.2491  -0.1943  -0.1600   3.1767  
## 
## Coefficients:
##             Estimate Std. Error z value Pr(>|z|)    
## (Intercept) -6.78108    1.15148  -5.889 3.89e-09 ***
## rainmean     0.07236    0.02377   3.045  0.00233 ** 
## ---
## Signif. codes:  0 '***' 0.001 '**' 0.01 '*' 0.05 '.' 0.1 ' ' 1
## 
## (Dispersion parameter for binomial family taken to be 1)
## 
##     Null deviance: 148.08  on 609  degrees of freedom
## Residual deviance: 139.44  on 608  degrees of freedom
## AIC: 143.44
## 
## Number of Fisher Scoring iterations: 7
## 
## 
## Call:
## glm(formula = outbreak ~ rainmean, family = binomial, data = I[which(I$Year %in% 
##     c(1970:2020)), ])
## 
## Deviance Residuals: 
##     Min       1Q   Median       3Q      Max  
## -0.6484  -0.2452  -0.1959  -0.1620   3.1915  
## 
## Coefficients:
##             Estimate Std. Error z value Pr(>|z|)    
## (Intercept) -6.22104    0.96312  -6.459 1.05e-10 ***
## rainmean     0.05858    0.01905   3.076   0.0021 ** 
## ---
## Signif. codes:  0 '***' 0.001 '**' 0.01 '*' 0.05 '.' 0.1 ' ' 1
## 
## (Dispersion parameter for binomial family taken to be 1)
## 
##     Null deviance: 148.08  on 609  degrees of freedom
## Residual deviance: 139.44  on 608  degrees of freedom
## AIC: 143.44
## 
## Number of Fisher Scoring iterations: 7
## 
## 
## Call:
## glm(formula = outbreak ~ rainmean, family = binomial, data = I[which(I$Year %in% 
##     c(1970:2020)), ])
## 
## Deviance Residuals: 
##     Min       1Q   Median       3Q      Max  
## -0.6477  -0.2455  -0.1958  -0.1632   3.1666  
## 
## Coefficients:
##             Estimate Std. Error z value Pr(>|z|)    
## (Intercept) -6.01829    0.90283  -6.666 2.63e-11 ***
## rainmean     0.05373    0.01757   3.059  0.00222 ** 
## ---
## Signif. codes:  0 '***' 0.001 '**' 0.01 '*' 0.05 '.' 0.1 ' ' 1
## 
## (Dispersion parameter for binomial family taken to be 1)
## 
##     Null deviance: 148.08  on 609  degrees of freedom
## Residual deviance: 139.59  on 608  degrees of freedom
## AIC: 143.59
## 
## Number of Fisher Scoring iterations: 7
## 
## 
## Call:
## glm(formula = outbreak ~ rainmean, family = binomial, data = I[which(I$Year %in% 
##     c(1970:2020)), ])
## 
## Deviance Residuals: 
##     Min       1Q   Median       3Q      Max  
## -0.5655  -0.2478  -0.1972  -0.1628   3.2089  
## 
## Coefficients:
##             Estimate Std. Error z value Pr(>|z|)    
## (Intercept) -7.00255    1.24993  -5.602 2.11e-08 ***
## rainmean     0.07814    0.02635   2.965  0.00302 ** 
## ---
## Signif. codes:  0 '***' 0.001 '**' 0.01 '*' 0.05 '.' 0.1 ' ' 1
## 
## (Dispersion parameter for binomial family taken to be 1)
## 
##     Null deviance: 148.08  on 609  degrees of freedom
## Residual deviance: 139.87  on 608  degrees of freedom
## AIC: 143.87
## 
## Number of Fisher Scoring iterations: 7
## 
## 
## Call:
## glm(formula = outbreak ~ rainmean, family = binomial, data = I[which(I$Year %in% 
##     c(1970:2020)), ])
## 
## Deviance Residuals: 
##     Min       1Q   Median       3Q      Max  
## -0.5944  -0.2500  -0.1994  -0.1640   3.1873  
## 
## Coefficients:
##             Estimate Std. Error z value Pr(>|z|)    
## (Intercept) -6.91329    1.22598  -5.639 1.71e-08 ***
## rainmean     0.07600    0.02577   2.949  0.00318 ** 
## ---
## Signif. codes:  0 '***' 0.001 '**' 0.01 '*' 0.05 '.' 0.1 ' ' 1
## 
## (Dispersion parameter for binomial family taken to be 1)
## 
##     Null deviance: 148.08  on 609  degrees of freedom
## Residual deviance: 139.95  on 608  degrees of freedom
## AIC: 143.95
## 
## Number of Fisher Scoring iterations: 7
## 
## 
## Call:
## glm(formula = outbreak ~ rainmean, family = binomial, data = I[which(I$Year %in% 
##     c(1970:2020)), ])
## 
## Deviance Residuals: 
##     Min       1Q   Median       3Q      Max  
## -0.5543  -0.2492  -0.1968  -0.1627   3.1377  
## 
## Coefficients:
##             Estimate Std. Error z value Pr(>|z|)    
## (Intercept) -6.66302    1.14693  -5.809 6.27e-09 ***
## rainmean     0.06987    0.02379   2.937  0.00332 ** 
## ---
## Signif. codes:  0 '***' 0.001 '**' 0.01 '*' 0.05 '.' 0.1 ' ' 1
## 
## (Dispersion parameter for binomial family taken to be 1)
## 
##     Null deviance: 148.08  on 609  degrees of freedom
## Residual deviance: 140.05  on 608  degrees of freedom
## AIC: 144.05
## 
## Number of Fisher Scoring iterations: 7
## 
## 
## Call:
## glm(formula = outbreak ~ rainmean, family = binomial, data = I[which(I$Year %in% 
##     c(1970:2020)), ])
## 
## Deviance Residuals: 
##     Min       1Q   Median       3Q      Max  
## -0.5434  -0.2487  -0.1978  -0.1630   3.1323  
## 
## Coefficients:
##             Estimate Std. Error z value Pr(>|z|)    
## (Intercept) -6.73353    1.17225  -5.744 9.24e-09 ***
## rainmean     0.07166    0.02445   2.930  0.00339 ** 
## ---
## Signif. codes:  0 '***' 0.001 '**' 0.01 '*' 0.05 '.' 0.1 ' ' 1
## 
## (Dispersion parameter for binomial family taken to be 1)
## 
##     Null deviance: 148.08  on 609  degrees of freedom
## Residual deviance: 140.09  on 608  degrees of freedom
## AIC: 144.09
## 
## Number of Fisher Scoring iterations: 7
## 
## 
## Call:
## glm(formula = outbreak ~ rainmean, family = binomial, data = I[which(I$Year %in% 
##     c(1970:2020)), ])
## 
## Deviance Residuals: 
##     Min       1Q   Median       3Q      Max  
## -0.6279  -0.2425  -0.1982  -0.1665   3.1724  
## 
## Coefficients:
##             Estimate Std. Error z value Pr(>|z|)    
## (Intercept) -5.77816    0.84554  -6.834 8.28e-12 ***
## rainmean     0.04816    0.01621   2.970  0.00297 ** 
## ---
## Signif. codes:  0 '***' 0.001 '**' 0.01 '*' 0.05 '.' 0.1 ' ' 1
## 
## (Dispersion parameter for binomial family taken to be 1)
## 
##     Null deviance: 148.08  on 609  degrees of freedom
## Residual deviance: 140.11  on 608  degrees of freedom
## AIC: 144.11
## 
## Number of Fisher Scoring iterations: 7
## 
## 
## Call:
## glm(formula = outbreak ~ rainmean, family = binomial, data = I[which(I$Year %in% 
##     c(1970:2020)), ])
## 
## Deviance Residuals: 
##     Min       1Q   Median       3Q      Max  
## -0.6320  -0.2456  -0.1969  -0.1654   3.1868  
## 
## Coefficients:
##             Estimate Std. Error z value Pr(>|z|)    
## (Intercept) -5.94413    0.90070  -6.599 4.13e-11 ***
## rainmean     0.05222    0.01762   2.964  0.00304 ** 
## ---
## Signif. codes:  0 '***' 0.001 '**' 0.01 '*' 0.05 '.' 0.1 ' ' 1
## 
## (Dispersion parameter for binomial family taken to be 1)
## 
##     Null deviance: 148.08  on 609  degrees of freedom
## Residual deviance: 140.12  on 608  degrees of freedom
## AIC: 144.12
## 
## Number of Fisher Scoring iterations: 7
## 
## 
## Call:
## glm(formula = outbreak ~ rainmean, family = binomial, data = I[which(I$Year %in% 
##     c(1970:2020)), ])
## 
## Deviance Residuals: 
##     Min       1Q   Median       3Q      Max  
## -0.5643  -0.2522  -0.1997  -0.1640   3.1062  
## 
## Coefficients:
##             Estimate Std. Error z value Pr(>|z|)    
## (Intercept) -6.48606    1.11026  -5.842 5.16e-09 ***
## rainmean     0.06576    0.02296   2.864  0.00418 ** 
## ---
## Signif. codes:  0 '***' 0.001 '**' 0.01 '*' 0.05 '.' 0.1 ' ' 1
## 
## (Dispersion parameter for binomial family taken to be 1)
## 
##     Null deviance: 148.08  on 609  degrees of freedom
## Residual deviance: 140.46  on 608  degrees of freedom
## AIC: 144.46
## 
## Number of Fisher Scoring iterations: 7
## 
## 
## Call:
## glm(formula = outbreak ~ rainmean, family = binomial, data = I[which(I$Year %in% 
##     c(1970:2020)), ])
## 
## Deviance Residuals: 
##     Min       1Q   Median       3Q      Max  
## -0.5717  -0.2447  -0.2001  -0.1668   3.1324  
## 
## Coefficients:
##             Estimate Std. Error z value Pr(>|z|)    
## (Intercept) -6.20916    1.01578  -6.113  9.8e-10 ***
## rainmean     0.05894    0.02059   2.862   0.0042 ** 
## ---
## Signif. codes:  0 '***' 0.001 '**' 0.01 '*' 0.05 '.' 0.1 ' ' 1
## 
## (Dispersion parameter for binomial family taken to be 1)
## 
##     Null deviance: 148.08  on 609  degrees of freedom
## Residual deviance: 140.54  on 608  degrees of freedom
## AIC: 144.54
## 
## Number of Fisher Scoring iterations: 7
## 
## 
## Call:
## glm(formula = outbreak ~ rainmean, family = binomial, data = I[which(I$Year %in% 
##     c(1970:2020)), ])
## 
## Deviance Residuals: 
##     Min       1Q   Median       3Q      Max  
## -0.6047  -0.2430  -0.1980  -0.1699   3.1087  
## 
## Coefficients:
##             Estimate Std. Error z value Pr(>|z|)    
## (Intercept) -5.53649    0.78888  -7.018 2.25e-12 ***
## rainmean     0.04260    0.01489   2.861  0.00422 ** 
## ---
## Signif. codes:  0 '***' 0.001 '**' 0.01 '*' 0.05 '.' 0.1 ' ' 1
## 
## (Dispersion parameter for binomial family taken to be 1)
## 
##     Null deviance: 148.08  on 609  degrees of freedom
## Residual deviance: 140.72  on 608  degrees of freedom
## AIC: 144.72
## 
## Number of Fisher Scoring iterations: 6
## 
## 
## Call:
## glm(formula = outbreak ~ rainmean, family = binomial, data = I[which(I$Year %in% 
##     c(1970:2020)), ])
## 
## Deviance Residuals: 
##     Min       1Q   Median       3Q      Max  
## -0.5790  -0.2405  -0.1978  -0.1710   3.0989  
## 
## Coefficients:
##             Estimate Std. Error z value Pr(>|z|)    
## (Intercept) -5.38507    0.73944  -7.283 3.27e-13 ***
## rainmean     0.03890    0.01361   2.858  0.00426 ** 
## ---
## Signif. codes:  0 '***' 0.001 '**' 0.01 '*' 0.05 '.' 0.1 ' ' 1
## 
## (Dispersion parameter for binomial family taken to be 1)
## 
##     Null deviance: 148.08  on 609  degrees of freedom
## Residual deviance: 140.75  on 608  degrees of freedom
## AIC: 144.75
## 
## Number of Fisher Scoring iterations: 6
## 
## 
## Call:
## glm(formula = outbreak ~ rainmean, family = binomial, data = I[which(I$Year %in% 
##     c(1970:2020)), ])
## 
## Deviance Residuals: 
##     Min       1Q   Median       3Q      Max  
## -0.6077  -0.2438  -0.2006  -0.1692   3.0828  
## 
## Coefficients:
##             Estimate Std. Error z value Pr(>|z|)    
## (Intercept) -5.68619    0.84223  -6.751 1.46e-11 ***
## rainmean     0.04631    0.01627   2.846  0.00443 ** 
## ---
## Signif. codes:  0 '***' 0.001 '**' 0.01 '*' 0.05 '.' 0.1 ' ' 1
## 
## (Dispersion parameter for binomial family taken to be 1)
## 
##     Null deviance: 148.08  on 609  degrees of freedom
## Residual deviance: 140.77  on 608  degrees of freedom
## AIC: 144.77
## 
## Number of Fisher Scoring iterations: 6
## 
## 
## Call:
## glm(formula = outbreak ~ rainmean, family = binomial, data = I[which(I$Year %in% 
##     c(1970:2020)), ])
## 
## Deviance Residuals: 
##     Min       1Q   Median       3Q      Max  
## -0.5888  -0.2463  -0.2000  -0.1676   3.1046  
## 
## Coefficients:
##             Estimate Std. Error z value Pr(>|z|)    
## (Intercept) -6.01061    0.95652  -6.284  3.3e-10 ***
## rainmean     0.05424    0.01917   2.830  0.00466 ** 
## ---
## Signif. codes:  0 '***' 0.001 '**' 0.01 '*' 0.05 '.' 0.1 ' ' 1
## 
## (Dispersion parameter for binomial family taken to be 1)
## 
##     Null deviance: 148.08  on 609  degrees of freedom
## Residual deviance: 140.77  on 608  degrees of freedom
## AIC: 144.77
## 
## Number of Fisher Scoring iterations: 6
## 
## 
## Call:
## glm(formula = outbreak ~ rainmean, family = binomial, data = I[which(I$Year %in% 
##     c(1970:2020)), ])
## 
## Deviance Residuals: 
##     Min       1Q   Median       3Q      Max  
## -0.5572  -0.2498  -0.2008  -0.1665   3.1431  
## 
## Coefficients:
##             Estimate Std. Error z value Pr(>|z|)    
## (Intercept) -6.66251    1.19544  -5.573  2.5e-08 ***
## rainmean     0.07038    0.02520   2.793  0.00523 ** 
## ---
## Signif. codes:  0 '***' 0.001 '**' 0.01 '*' 0.05 '.' 0.1 ' ' 1
## 
## (Dispersion parameter for binomial family taken to be 1)
## 
##     Null deviance: 148.08  on 609  degrees of freedom
## Residual deviance: 140.81  on 608  degrees of freedom
## AIC: 144.81
## 
## Number of Fisher Scoring iterations: 7
## 
## 
## Call:
## glm(formula = outbreak ~ rainmean, family = binomial, data = I[which(I$Year %in% 
##     c(1970:2020)), ])
## 
## Deviance Residuals: 
##     Min       1Q   Median       3Q      Max  
## -0.6049  -0.2431  -0.2006  -0.1697   3.1500  
## 
## Coefficients:
##             Estimate Std. Error z value Pr(>|z|)    
## (Intercept) -5.67401    0.84225  -6.737 1.62e-11 ***
## rainmean     0.04606    0.01629   2.827   0.0047 ** 
## ---
## Signif. codes:  0 '***' 0.001 '**' 0.01 '*' 0.05 '.' 0.1 ' ' 1
## 
## (Dispersion parameter for binomial family taken to be 1)
## 
##     Null deviance: 148.08  on 609  degrees of freedom
## Residual deviance: 140.87  on 608  degrees of freedom
## AIC: 144.87
## 
## Number of Fisher Scoring iterations: 6
## 
## 
## Call:
## glm(formula = outbreak ~ rainmean, family = binomial, data = I[which(I$Year %in% 
##     c(1970:2020)), ])
## 
## Deviance Residuals: 
##     Min       1Q   Median       3Q      Max  
## -0.5241  -0.2483  -0.2008  -0.1680   3.1719  
## 
## Coefficients:
##             Estimate Std. Error z value Pr(>|z|)    
## (Intercept) -6.75721    1.24454  -5.429 5.65e-08 ***
## rainmean     0.07282    0.02648   2.750  0.00596 ** 
## ---
## Signif. codes:  0 '***' 0.001 '**' 0.01 '*' 0.05 '.' 0.1 ' ' 1
## 
## (Dispersion parameter for binomial family taken to be 1)
## 
##     Null deviance: 148.08  on 609  degrees of freedom
## Residual deviance: 141.04  on 608  degrees of freedom
## AIC: 145.04
## 
## Number of Fisher Scoring iterations: 7
## 
## 
## Call:
## glm(formula = outbreak ~ rainmean, family = binomial, data = I[which(I$Year %in% 
##     c(1970:2020)), ])
## 
## Deviance Residuals: 
##     Min       1Q   Median       3Q      Max  
## -0.5462  -0.2498  -0.2001  -0.1673   3.0814  
## 
## Coefficients:
##             Estimate Std. Error z value Pr(>|z|)    
## (Intercept) -6.46194    1.14030  -5.667 1.45e-08 ***
## rainmean     0.06559    0.02387   2.748    0.006 ** 
## ---
## Signif. codes:  0 '***' 0.001 '**' 0.01 '*' 0.05 '.' 0.1 ' ' 1
## 
## (Dispersion parameter for binomial family taken to be 1)
## 
##     Null deviance: 148.08  on 609  degrees of freedom
## Residual deviance: 141.05  on 608  degrees of freedom
## AIC: 145.05
## 
## Number of Fisher Scoring iterations: 7
## 
## 
## Call:
## glm(formula = outbreak ~ rainmean, family = binomial, data = I[which(I$Year %in% 
##     c(1970:2020)), ])
## 
## Deviance Residuals: 
##     Min       1Q   Median       3Q      Max  
## -0.5624  -0.2504  -0.2024  -0.1691   3.1538  
## 
## Coefficients:
##             Estimate Std. Error z value Pr(>|z|)    
## (Intercept) -6.68347    1.22163  -5.471 4.48e-08 ***
## rainmean     0.07101    0.02591   2.741  0.00613 ** 
## ---
## Signif. codes:  0 '***' 0.001 '**' 0.01 '*' 0.05 '.' 0.1 ' ' 1
## 
## (Dispersion parameter for binomial family taken to be 1)
## 
##     Null deviance: 148.08  on 609  degrees of freedom
## Residual deviance: 141.09  on 608  degrees of freedom
## AIC: 145.09
## 
## Number of Fisher Scoring iterations: 7
## 
## 
## Call:
## glm(formula = outbreak ~ rainmean, family = binomial, data = I[which(I$Year %in% 
##     c(1970:2020)), ])
## 
## Deviance Residuals: 
##     Min       1Q   Median       3Q      Max  
## -0.5468  -0.2524  -0.2016  -0.1672   3.0747  
## 
## Coefficients:
##             Estimate Std. Error z value Pr(>|z|)    
## (Intercept) -6.35701    1.10565  -5.750 8.95e-09 ***
## rainmean     0.06303    0.02301   2.739  0.00615 ** 
## ---
## Signif. codes:  0 '***' 0.001 '**' 0.01 '*' 0.05 '.' 0.1 ' ' 1
## 
## (Dispersion parameter for binomial family taken to be 1)
## 
##     Null deviance: 148.08  on 609  degrees of freedom
## Residual deviance: 141.11  on 608  degrees of freedom
## AIC: 145.11
## 
## Number of Fisher Scoring iterations: 7
```

```
I$fit <- I$fit/TOP  #to get the average fitted values across the top models
```

## Overview plot of model results

For each model the AIC was calculated and we classified as the **top models** the models that where within 2 AIC units of the model with the minimal AIC.

Below a plot of the AIC values is presented with combinations marked with a white X identifying the **top models**.

```
P <- ggplot(data=M, aes(x=RainfallPeriod,y=TimeLagPeriod, fill=AIC))+
  geom_tile() +
  theme_classic() +
  geom_text(aes(x=RainfallPeriod, y=TimeLagPeriod, label = top), color = "white", size = 3) +
  theme(aspect.ratio=1, legend.position="top", legend.text=element_text(angle=90,vjust = 0.5, hjust=0.5),
    legend.direction = "horizontal") +
  labs(y = "Time-lag period (months)",
       x = "Rainfall period (months)")
plot(P)
```

```
png("plot MDB rain and outbreaks AIC.png", width = 120, height = 90, units='mm', res = 600)
print(P)
dev.off()
```

```
## png 
##   2
```

## Same plot with coefficient

```
P <- ggplot(data=M, aes(x=RainfallPeriod,y=TimeLagPeriod, fill=coef))+
  geom_tile() +
  theme_classic() +
  geom_text(aes(x=RainfallPeriod, y=TimeLagPeriod, label = top), color = "white", size = 3) +
  theme(aspect.ratio=1,legend.position="top", legend.text=element_text(angle=90,vjust = 0.5, hjust=0.5),
    legend.direction = "horizontal") +
  scale_fill_gradient2(low = "white", high = "red") +
  labs(y = "Time-lag period (months)",
       x = "Rainfall period (months)")
plot(P)
```

```
png("plot MDB rain and outbreaks coef.png", width = 120, height = 90, units='mm', res = 600)
print(P)
dev.off()
```

```
## png 
##   2
```

## Rainfall and outbreaks with 12 month running mean

Monthly rainfall data is plotted in grey.

A right alligned rolling mean is plotted in blue, which means that it indicates the mean rainfall in the preceding 12 months.

Outbreaks are plotted as red triangles

The orange line is an index of outbreak probability based on the average predicted probability of the top models falling within 2 AIC units.

```
#calculate year value
I$Date <- I$Year+I$month/12

#add fitted values from best model as Expected Probabilities
I$ExpProb <- 1 / (1 + exp(-I$fit))

jitter <- position_jitter(width = 0, height = 5)
P <- ggplot(data=I, aes(x=Date)) +
  theme_classic() +
  geom_line(aes(y=rainfall),color="grey") +
  geom_line(aes(y=rollmean(rainfall, 12, na.pad=TRUE,align="right")), color="blue")+
  #geom_line(aes(y=rollmean(rainfall, 12, na.pad=TRUE,align="center")), color="green")+
  xlim(1970,2021) +
  labs(y = "Rainfall (mm)",
       x = "")+
  #geom_line(aes(y=ExpProb*1000-400), color="orange") +
  geom_point(data=O, aes(x=Year+month/12, y=20), position=jitter, shape=2, color="red", size=2.5)
plot(P)
```

```
png("plot MDB rain and outbreaks.png", width = 120, height = 100, units='mm', res = 600)
print(P)
dev.off()
```

```
## png 
##   2
```
